# Identifying the Perceived Severity of Patient-Generated Telemedical Queries Regarding COVID: Developing and Evaluating a Transfer Learning–Based Solution

**DOI:** 10.2196/37770

**Published:** 2022-09-02

**Authors:** Joseph Gatto, Parker Seegmiller, Garrett Johnston, Sarah Masud Preum

**Affiliations:** 1 Department of Computer Science Dartmouth College Hanover, NH United States

**Keywords:** natural language processing, transfer learning, telemedicine triage, COVID-19, health resource, health care, patient query, learning solution, telemedical, lexical model, machine learning, BERT, telehealth

## Abstract

**Background:**

Triage of textual telemedical queries is a safety-critical task for medical service providers with limited remote health resources. The prioritization of patient queries containing medically severe text is necessary to optimize resource usage and provide care to those with time-sensitive needs.

**Objective:**

We aim to evaluate the effectiveness of transfer learning solutions on the task of telemedical triage and provide a thorough error analysis, identifying telemedical queries that challenge state-of-the-art natural language processing (NLP) systems. Additionally, we aim to provide a publicly available telemedical query data set with labels for severity classification for telemedical triage of respiratory issues.

**Methods:**

We annotated 573 medical queries from 3 online health platforms: HealthTap, HealthcareMagic, and iCliniq. We then evaluated 6 transfer learning solutions utilizing various text-embedding strategies. Specifically, we first established a baseline using a lexical classification model with term frequency–inverse document frequency (TF-IDF) features. Next, we investigated the effectiveness of global vectors for text representation (GloVe), a pretrained word-embedding method. We evaluated the performance of GloVe embeddings in the context of support vector machines (SVMs), bidirectional long short-term memory (bi-LSTM) networks, and hierarchical attention networks (HANs). Finally, we evaluated the performance of contextual text embeddings using transformer-based architectures. Specifically, we evaluated bidirectional encoder representation from transformers (BERT), Bio+Clinical-BERT, and Sentence-BERT (SBERT) on the telemedical triage task.

**Results:**

We found that a simple lexical model achieved a mean F1 score of 0.865 (SD 0.048) on the telemedical triage task. GloVe-based models using SVMs, HANs, and bi-LSTMs achieved a 0.8-, 1.5-, and 2.1-point increase in the F1 score, respectively. Transformer-based models, such as BERT, Bio+Clinical-BERT, and SBERT, achieved a mean F1 score of 0.914 (SD 0.034), 0.904 (SD 0.041), and 0.917 (SD 0.037), respectively. The highest-performing model, SBERT, provided a statistically significant improvement compared to all GloVe-based and lexical baselines. However, no statistical significance was found when comparing transformer-based models. Furthermore, our error analysis revealed highly challenging query types, including those with complex negations, temporal relationships, and patient intents.

**Conclusions:**

We showed that state-of-the-art transfer learning techniques work well on the telemedical triage task, providing significant performance increase over lexical models. Additionally, we released a public telemedical triage data set using labeled questions from online medical question-and-answer (Q&A) platforms. Our analysis highlights various avenues for future works that explicitly model such query challenges.

## Introduction

### Background

The COVID-19 pandemic has led to an increased demand for telemedicine services [1]. Projections state that up to 50% of consultations could be performed through telehealth by 2025 for certain demographic groups [2]. The general demographic makeup of telemedicine patients, 1 study finds, is most often White English-speaking females using private medical insurance, with minority groups using significantly less telemedical services [3]. Patients use these services to communicate with a diverse set of medical specialists, including dentists, rheumatologists, and prenatal care specialists, among many others, all with high levels of satisfaction [2]. These studies, however, examine patients who utilize telemedicine services to interact with their existing care providers in a remote setting. Recently, there has been a rise in affordable and accessible telemedicine platforms that connect anyone with an internet connection to licensed medical professionals worldwide. Such platforms include *HealthTap* [4], *iCliniq*, and *HealthcareMagic*. HealthTap, for example, is a Health Insurance Portability and Accountability Act (HIPAA)–certified website that provides online users with access to a qualified doctor with an active US medical license [4]. These platforms are easy to access and provide greater accessibility to professional medical consultation. However, such ease and accessibility can cause these services to be flooded with questions that are *not* medically severe or relevant. This is a safety-critical problem, as an abundance of nonsevere medical queries will hinder the speed at which medical professionals can respond to time-sensitive issues. Evidence of this phenomenon was observed with COVID-19 hotlines, where confusion about coronavirus caused long wait times, with Margolius et al [5] finding that of the 12,512 calls made to their triage system between March 13 and April 20, 2020, “52% were not COVID-19 related or required no additional care.” Large numbers of nonsevere telemedical queries not only are dangerous but also may unnecessarily increase health care spending, as telemedical service convenience may encourage patients to inquire about negligible health concerns [6].

This situation necessitates a system for prioritizing which queries require immediate care. To address this problem, we examined data from 3 telemedicine platforms: HealthTap, iCliniq, and HealthcareMagic. These platforms facilitate written medical queries to be answered remotely by licensed doctors. Our goal was to optimize the time spent by health care workers by ranking patient queries by severity so that potentially severe queries are answered first. In this study, a query was deemed severe when a patient had at least 1 active COVID-19– or pneumonia-related symptom. Nonsevere queries, however, were from patients with no active symptoms who submitted general information requests, nonsensical text, or extremely vague questions. Telemedical triage conserves the limited amount of professional health care provider resources available to telemedicine platforms by prioritizing severe queries, encouraging remote medical care to be provided to those most desperately in need.

In this work, we examined telemedical triage through the lens of online medical question-and-answer (Q&A) forums. Specifically, we formulated triage as a binary text classification problem, where we aimed to classify medical queries [7] as either severe or not severe. We noted that this formulation does not capture the full spectrum of severity but is useful for lowering the priority of queries with no medical urgency. To do so, we introduced an extension to the publicly available data set *COVID-Dialogue* [8], which contains 603 doctor-patient conversations extracted from HealthTap, iCliniq, and HealthcareMagic, with labels for severe query classification. Given the limited number of available samples, we then investigated various transfer learning approaches to text classification and contrasted them with a lexical approach. Specifically, we explored the applicability of different embedding methods, such as global vectors for text representation (GloVe) and transformers, pretrained using both general and medical texts, in comparison to term frequency–inverse document frequency (TF-IDF) features. Our experiments showed that transformer-based solutions are a superior transfer learning approach for identifying severe medical queries. Additionally, we found that pretraining on medical texts provides no benefit when classifying our telemedical triage data set. Finally, we provided an in-depth error analysis of sentence bidirectional encoder representation from transformers (sentence-BERT or SBERT) [9], identifying challenging patient query patterns that motivate future work on telemedical triage. Specifically, we noticed difficulties in modeling the negation, temporality, and intent of symptom mentions in patient-generated textual queries.

Our contributions are as follows:

- We established baseline results across 6 relevant natural language processing (NLP) models on the telemedical triage task—identifying optimal pretraining strategies for query ranking according to severity. We identified contextual embedding models to work best for triage, with all transformer-based approaches achieving statistically significant improvements over both lexical and word embedding–based approaches. We found no benefit of pretraining transformer models with clinical text.
- We provided a thorough error analysis of SBERT and identified several medical query types that pose difficulties to NLP systems—where the core challenge is identified as the modeling of complex symptom presentations.
- To the best of our knowledge, we have provided the first publicly available telemedical triage classification data set using real samples from online telemedical services. All code and data for this study have been made publicly available [7].

### Related Works

#### Transfer Learning for Medical Text Classification

The need for data privacy and patient anonymity makes large-scale collection and labeling of health care texts extremely difficult. This has motivated the use of transfer learning in medical NLP to alleviate the challenges in resource-constrained modeling. In recent years, transfer learning has benefited greatly from leveraging large amounts of unlabeled text to train transformer-based models [10] using a masked language modeling objective. This framework allows common linguistic pattern understanding to be transferred to other downstream tasks, reducing the need for large amounts of labeled data to solve various low-resource problems.

Bidirectional encoder representation from transformers (BERT) [11] is a popular transformer-based model that has been pretrained on general text, such as the Wikipedia and Brown corpus. Medical inference tasks that require domain-specific linguistic knowledge, such as medical natural language inference [12] or medical concept extraction [13], have been shown to benefit significantly from pretraining on medical-specific text [14]. In this study, we explored transfer learning using both general and medical text as pretraining methods. Analysis of optimal pretraining strategies for telemedical triage is of interest as patient queries are typically composed of common language but often interwoven with complex medical terminology.

#### Machine Learning for Triage

The COVID-19 pandemic overwhelmed the US health care system, bringing about a demand for machine learning solutions for the telemedicine triage problem. For example, Lai et al [15] found that COVID-19 hotlines became overwhelmed with calls, prompting the development of an artificial intelligence (AI) system of patient ranking. Lai et al [15] used an AI chatbot to prescreen callers by asking questions that reveal whether a patient has COVID-19 symptoms. The resulting information is fed to a logic-based inference model, which determines whether further consultation from the hotline’s health care workers is required.

Hospital emergency departments (EDs) have similarly become overwhelmed with patients [16], causing dangerous treatment delays for those coming to the ED with an urgent need. Yao et al [16] trained a deep learning model to predict which patients will eventually require hospitalization by using incoming patient emergency medical records. This allows for an automated system of patient care prioritization that does not depend on nursing resources. Similar work was done by Gligorijevic et al [17], where a deep attention model was used to triage patients using multimodal electronic health record (EHR) data, where triage was formulated as a classification problem based on the Emergency Severity Index [18].

Unlike the aforementioned work, we viewed triage solely through the lens of textual queries—specifically those submitted by patients to telemedicine platforms. With rising demand for textual medical support, through either public medical Q&A platforms, such as HealthTap, or private doctor-patient messaging apps, we foresee a growing need for NLP solutions for the triage problem over free-text patient queries.

#### Medical Risk Identification

A similar work for triage of telemedicine platform messages was performed by Si et al [19], who classified doctor-patient messages based on their urgency by using data collected from adults at a university hospital. The data are unfortunately not public. Additionally, the message content contains queries about cardiology from patients to their existing cardiologists. This is in stark contrast to our data set, which is public, has a different label space, and contains samples about respiratory illnesses from patients to doctors they have never spoken to before (and thus are unable to make decisions using prior knowledge of medical history). Both our work and that of Si et al [19], however, explore BERT-based solutions to triage.

Additional, similar work resides in the realm of medical risk identification from text. For example, Fu et al [20] introduced a knowledge graph–based distant supervision approach to suicide risk prediction from social media posts, Wang et al [21] explored transformer-based solutions for depression risk prediction from social media data, and Klein et al [22] applied a BERT-based classifier to identify potential COVID-19 cases from tweets.

This work is similar in that we explored BERT-based solutions for medical risk identification. However, unlike social media data, medical queries submitted to telemedicine platforms often contain complex clinical terminology. Furthermore, the telemedical services through which doctors interact with patients contain less restrictive character limits, requiring modeling of long-range textual dependencies. Finally, social media–based studies have the luxury of large-scale data mining. In this study, we operated in an extremely resource-constrained data setting, which challenged our capacity to model and understand medical query text.

## Methods

### Data Set

In this study, we utilized the publicly available COVID-Dialogue data set [8]. This data set contains 603 anonymized patient queries extracted from 3 telemedicine platforms, namely HealthTap, iCliniq, and HealthcareMagic. The original data set was collected with the intention to facilitate better AI dialogue systems during the COVID-19 pandemic. Thus, each of the 603 doctor-patient conversations includes the full patient query, a summarized patient query, and the doctor’s response. The data set was not curated for text classification; thus, after filtering samples unusable in our classification setting (ie, duplicate, non-English, and out-of-scope entries—where “out of scope” is defined as entries not regarding COVID-19 or pneumonia symptoms), we annotated 573 (95%) samples. All multiturn dialogues were truncated to the initial patient utterance, and no doctor responses were used in our pipeline.

Each sample in the COVID-Dialogue data set contains queries regarding either COVID-19 or related pneumonia symptoms. Each sample includes no patient demographics or medical history; thus, severity was detected solely using a single free-text inquiry. Table 1 provides an example from each class in our labeled data set. The general goal of our labeling schema was to prioritize those with active symptoms and reduce the priority of the hundreds of samples that exhibit no medically severe text. Our final data set contained 314 (55%) severe samples and 259 (45%) nonsevere samples.

**Table 1:** Samples from the COVID-Dialogue data set with our introduced severity label. Nonsevere samples are often irrelevant queries or from patients with little to no symptoms. Severe samples always contain patients with active symptoms that may require medical attention.

Patient query	Ground truth label
“Should I shave my beard to reduce my chances of contracting coronavirus/covid-19?”	Not severe
“My daughter is 11 years old she has has pneumonia she has been sick since January 3rd symptoms keep changing. she is up at night itching all over her upper torso, head, and ears. She has major headache and abdominal pain.”	Severe

### Ethical Considerations

Given that the data are publicly available, no Institutional Review Board approval was required for this study.

The data set used for the development and evaluation of the solution is anonymous and does not reveal the identity of doctors and patients. No demographic information is available for this data set.

We consulted 3 professional health care providers about the real-world implications of such a telemedicine triage system. We consulted Drs Timothy E. Burdick, Stephen K. Liu, and Jiazuo H. Feng. All of them serve as primary care providers at a local teaching hospital. An interesting question regarding the ethical use of future telemedical triage systems is whether to include demographic, socioeconomic, physiological, or other EHR information in future medical triage systems, given such information is available. Although demographic or past medical history (eg, age of the patient, pre-existing conditions) might be relevant to determine the actual severity of the patient’s query, sucn information can also introduce bias. Related works on telemedical triage, such as Si et al [19], similarly propose the use of demographic information in future work. Determining the fairness and equity of such systems would require additional exploration with additional ground truth from the user that is available in the EHR data, including but not limited to emergency visits, urgent care visits, and scheduling new appointments immediately after receiving a response from the care provider. This is out of scope of this paper. However, we are currently designing a study to investigate this question by using EHR data collected from a local hospital, as mentioned later.

### Data Sources

Next, we describe the sources used in the collection of the COVID-Dialogue data set [8], which was publicly released in March 2020. Samples in this data set were collected between February 7 and March 25, 2020.

#### HealthTap

Founded in 2010, HealthTap is a telemedicine platform that remotely connects patients with US licensed medical professionals for a variety of services, including virtual consultations and doctor-patient Q&A. According to Dahl [23], patients have had close to 1 billion questions answered on HealthTap. Additionally, HealthTap accepts over 100 insurance plans and employs doctors from over 140 specialties. HealthTap data in the COVID-Dialogue data set were collected from its medical Q&A forum.

#### iCliniq

iCliniq is a virtual hospital providing video, voice, and text chat medical services to patients worldwide. iCliniq works with more than 3500 licensed doctors internationally, covering over 80 medical specialties. Samples from iCliniq were drawn from its medical Q&A forum.

#### HealthcareMagic

Unlike Healthtap and iCliniq, HealthcareMagic is strictly an online medical Q&A forum. With over 18,000 doctors across 78 medical specialties, 1.7 million questions have been answered on HealthcareMagic.

### Annotation Details

Each sample in our data set was annotated by 3 of the authors as either severe or nonsevere. Use of authors as annotators for small-scale medical web information has been successful in other studies [24,25]. Each annotator has a college degree and an adequate level of health literacy and has invested significant time to educate themselves on the potential symptoms associated with the 2 illnesses observed in this data set. We noted that the use of nonmedical professionals limited the degree of granularity with which we could label this data set. However, the annotators carefully reviewed the response to the original query to determine potential severe queries. In addition, the annotators observed that there were a lot of irrelevant samples compared to those exhibiting significant symptoms. For example, the following query can be safely annotated as “not severe” since it does not warrant significant medical knowledge to answer “Where can I get a COVID-19 test?” This question can be answered using Google Search for most parts of the United States. We noted, however, that assuming internet search availability might bias annotation against those in rural, remote areas without reliable access to the internet and familiarity with a web search for health. However, those without access to Google Search or an intent to use a web search for health issues would be also less likely to rely on telemedicine services [2].

We additionally noted that there might be some samples where the perceived severity based on the query and the response from the medical professional would be different from the actual severity of the condition of the patient. However, since we do not have any ground truth from the actual user, such cases cannot be resolved. This motivated us to pursue future work in this direction by utilizing our collaboration with doctors in a local hospital, as reported in the Future Work section. In addition, we performed a thorough error analysis of the performance of our proposed solution and illustrated its strengths and limitations with respect to this annotated data set. The final annotation for each sample was the majority vote label from the 3 annotators. The interannotator agreement across all samples was 82%. Next, we detail the annotation schema for both nonsevere and severe samples.

#### Not Severe

The guiding principle behind a *nonsevere* annotation was a patient query that did not indicate an active symptom, an immediate need for diagnosis, or an immediate need for a medical response. This included queries that were unspecific or speculative. Examples selected from the data set and their nonsevere annotation rationale are listed next.

Where can I get a covid test?

This query does not indicate immediate danger, a need for diagnosis, or a need for a medical response. This query can also be served by Google Search and thus does not need feedback from a medical professional.

Will I have to be hospitalised if I get the virus, I have type 1 diabetes.

Although this query is medically valid and deserves a response, the need is not deemed immediate as the patient has no active symptoms.

#### Severe

A *severe* annotation was given to a patient who indicated an active symptom that may present danger to the patient, an immediate need for diagnosis, or an immediate need for a medical response. This included queries in which a patient listed current symptoms or demonstrated a need for actionable doctor advice. Examples selected from the data set and their severe annotation rationale are listed next.

My son is not feeling well. He has a very snotty nose, sore throat, occasional flemmy cough, uneasy stomach. He had a headache last night. No fever. Is it a common cold or must he be checked for Covid 19. Not travelled or been in contact with anyone?

This query describes symptoms that are consistent with COVID-19 and demonstrate a sufficient need for medical advice.

Preauricular lymph node on left side very tender, scalp on left side of head tender and hurts to touch, superficial parotid lymph node area on left side swollen and tender. Pain behind both ears. No injury. Came on suddenly, has been 1 day. Temp 100.1°.

This query contains a clear, immediate danger to the patient and requires a medical response.

### Transfer Learning Methods

#### Bidirectional Encoder Representation From Transformers

BERT is a state-of-the-art transformer-based model that leverages unlabeled text to produce contextualized language representations [11]. In this study, we used standard BERT for the text classification pipeline outlined by Devlin et al [11], where we first generated contextualized text features using a pretrained BERT model, followed by feeding the special CLS token to a linear classification head, which outputs the final query label. We explored BERT for telemedical triage, as BERT has been shown to be successful in related tasks, such as depression risk prediction [21], suicide risk prediction [20], and COVID-19 case identification [22].

#### Bio+Clinical-BERT

The Bio+Clinical-BERT architecture is the same as that of BERT but with weights pretrained on medical texts. Specifically, this pretraining procedure first takes the BioBERT model [26], which is BERT fine-tuned on biomedical research text collected from PubMed. Next, BioBERT is fine-tuned on clinical notes from the Medical Information Mart for Intensive Care (MIMIC)-III database [27], producing Bio+Clinical-BERT [14]. The combination of BERT, biomedical research texts, and clinical notes was shown to significantly outperform BERT on the medical natural language inference task [12]. Thus, we use Bio+Clinical-BERT as our medically informed architectural baseline for the patient query task. This is our only transfer learning approach leveraging knowledge from medical texts.

#### SBERT With Triplet Loss

We also explored the effectiveness of SBERT [9] for telemedical triage. Unlike BERT, which learns to output a contextualized embedding for *every* input token, SBERT produces a single embedding for a given input. SBERT has proved effective in adjacent medical NLP tasks, such as COVID-19 vaccine sentiment analysis [28] and COVID-19 misinformation detection [29]. In this study, we used SBERT as it permits both text classification and useful methods of embedding interpretability.

To perform text classification with SBERT, we first fine-tuned an SBERT model to minimize the following triplet loss function:




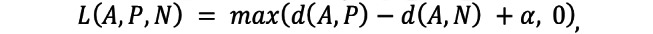




where A is an anchor sample, P is a positive sample (same class as A), N is a negative sample (opposite class of A) and d is the cosine-similarity distance function. This objective can be interpreted as learning to push query embeddings from the same class close together in embedding space, while pushing samples from opposite classes further apart. The margin parameter α influences the distance between positive and negative pairs in embedding space. To generate a training triple, a given sample was randomly paired with a sample from the same and opposite classes. This process was repeated 10 times per sample, generating 4580 training triplets.

Using the embeddings from the fine-tuned SBERT model, we then trained a K-nearest neighbor (KNN) classifier using the Scikit-Learn package [30]. Specifically, we set the number of neighbors K=10, otherwise using the default parameters provided by Scikit-Learn (which uses the Minkowski distance metric with p=2). The KNN was trained using the same training set as all other experiments and then used to label the test set queries based on their relationship to the training samples in the embedding space.

### Baseline Experiments

For TF-IDF+SVM, we fed the TF-IDF [31] feature vector from the patient query to the support vector machine (SVM) classifier [32]. This baseline examined the effectiveness of a simple lexical model on telemedical triage.

For GloVE+SVM, we obtained the pretrained GloVe [33] embedding for each word in the patient query and fed the mean vector to the SVM classifier. This baseline tested the performance of transfer learning without the use of contextual modeling.

A 2-layer bidirectional long short-term memory (bi-LSTM) model was trained on GloVe embeddings for classification. The bi-LSTM model examined the effectiveness of contextual sequence modeling on pretrained word embeddings. Bi-LSTM models have been shown to be effective in a variety of clinical text prediction tasks [34].

The hierarchical attention network (HAN) [35] for text classification mimics the natural language hierarchy by modeling attention at the sentence and word level for document classification. HAN word embeddings in this experiment were initialized using GloVe. Other prior works have established a HAN to be an effective classifier for medical text [36].

### Evaluation Setting

For each experiment, we reported the weighted mean F1, precision, and recall scores over a 5-fold cross-validation. Additionally, we reported the 95% CI for the reported mean. Finally, we conducted statistical significance testing using the McNemar test [37] for each model with respect to our top-performing approach, SBERT. Each train and test split contained approximately 458 and 115 samples, respectively.

## Results

In this section, we present our results of the telemedical triage task across various NLP baselines. Our goal is to answer the following research questions (RQs):

- How effective are transfer learning models for telemedical triage for COVID-19–related queries when compared to other text classification models?
- What types of health queries challenge state-of-the-art NLP systems?

### Analysis

Our results showed that telemedical triage benefits greatly from transfer learning as our lowest-performing model, TF-IDF+SVM, used no transfer learning. TF-IDF features achieved a reasonable mean F1 score of 0.865 (SD 0.048). However, we found a 0.8-, 1.5-, and 2.1-point increase in the F1 score by applying GloVE-based models, such as GloVe+SVM, HAN, and bi-LSTM models, respectively. Generally, light-weight modeling options, such as TF-IDF and GloVe, report reasonable F1 scores and are thus viable solutions in cases with limited computational resources.

#### RQ1: How Effective Are Transfer-Learning Models on Telemedical Triage ?

We found transformer-based models to be the superior method of transfer learning, with BERT, Bio+Clinical BERT, and SBERT achieving mean F1 scores of 0.914 (SD 0.034), 0.904 (SD 0.041), and 0.917 (SD 0.037), respectively (Table 2). We noted that Bio+Clinical-BERT did not outperform the BERT baseline. This is likely due to the difference in language found in BERT versus Bio+Clinical-BERT training data. Clinical notes used to train Bio+Clinical-BERT are written by medical practitioners and thus far more technical than queries written by patients. Thus, although telemedical query texts do contain medical terminology, clinical note pretraining is not helpful in this setting.

Our results showed that SBERT, on average, is the best predictor of query severity, producing both the highest average F1, precision, and recall scores compared to other approaches. A higher recall is particularly important in the realm of triage as reducing false negatives is more important in such a safety-critical task.

Using the McNemar test for statistical significance, we found SBERT to perform significantly better than TF-IDF+SVM (*P*<.001), GloVE+SVM (*P*=.001), bi-LSTM (*P*=.03), and HAN (*P*=.001). However, tests for statistical significance failed when comparing SBERT with other transformer-based models, such as BERT (*P*=.81) and Clinical-BERT (*P*=.22). Thus, the difference in predictive distributions between transformer-based approaches is insignificant, with all being valid options for transfer learning solutions to telemedical triage.

Although the performance of the transformer-based models was high (all F1 scores>0.9), it is important to note that the general problem of telemedical triage is far from solved. This study looked at triage through the narrow lens of COVID-19– and pneumonia-related queries; diseases involving nonrespiratory complications would not be recognized by this system.

**Table 2:** Results displaying triage performance for all models. Each metric is the mean result over 5-fold cross-validation, surrounded by the 95% CI computed using the metric score for each validation fold.

Model	F1 score, mean (SD)	Precision, mean (SD)	Recall, mean (SD)
TF-IDF^a^+SVM^b^	0.865 (0.048)	0.871 (0.043)	0.865 (0.048)
GloVe^c^+SVM	0.873 (0.036)	0.878 (0.030)	0.874 (0.035)
Bi-LSTM^d^	0.886 (0.051)	0.880 (0.049)	0.879 (0.052)
HAN^e^	0.880 (0.035)	0.890 (0.031)	0.880 (0.033)
BERT^f^	0.914 (0.034)	0.917 (0.033)	0.914 (0.034)
Bio+Clinical-BERT	0.904 (0.041)	0.905 (0.040)	0.904 (0.041)
SBERT^g^	0.917 (0.037)	0.920 (0.034)	0.918 (0.036)

^a^TF-IDF: term frequency–inverse document frequency.

^b^SVM: support vector machine.

^c^GloVe: global vectors for text representation.

^d^Bi-LSTM: bidirectional long short-term memory.

^e^HAN: hierarchical attention network.

^f^BERT: bidirectional encoder representation from transformers.

^g^SBERT: sentence bidirectional encoder representation from transformers.

#### RQ2: What Types of Telemedical Queries Challenge State-of-the-Art NLP Systems?

In the previous section, we identified transformer-based models to be the most effective form of pretraining for triage. To identify telemedical queries that are difficult to triage, we investigated SBERT, as this architecture outputs a single embedding for an entire query, which is useful for interpretability.

We first visualized the SBERT embedding for each test sample in 1 of our test splits using t-distributed stochastic neighbor embedding (t-SNE) [38], projecting the 768D SBERT embeddings for each sample down to a 2D space that aims to preserve the embedding distance found in higher dimensions.

**Figure 1:**
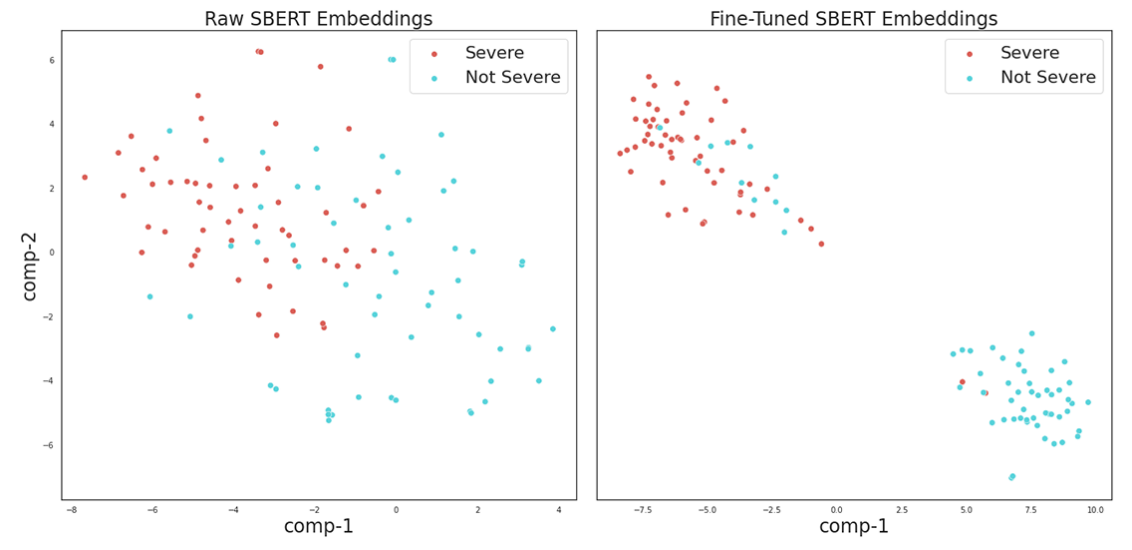
SBERT embeddings projected to 2 dimensions using t-SNE. The left image depicts how the test samples are distributed in the embedding space prior to triplet loss–based fine-tuning. The right image displays how SBERT learns to separate query embeddings in the embedding space. Note: The comp-1 and comp-2 axes denote the names of the 2 dimensions onto which t-SNE projects the 768D embeddings, where “comp” is short for “component”. SBERT: sentence bidirectional encoder representation from transformers; t-SNE: t-distributed stochastic neighbor embedding.

**Figure 2:**
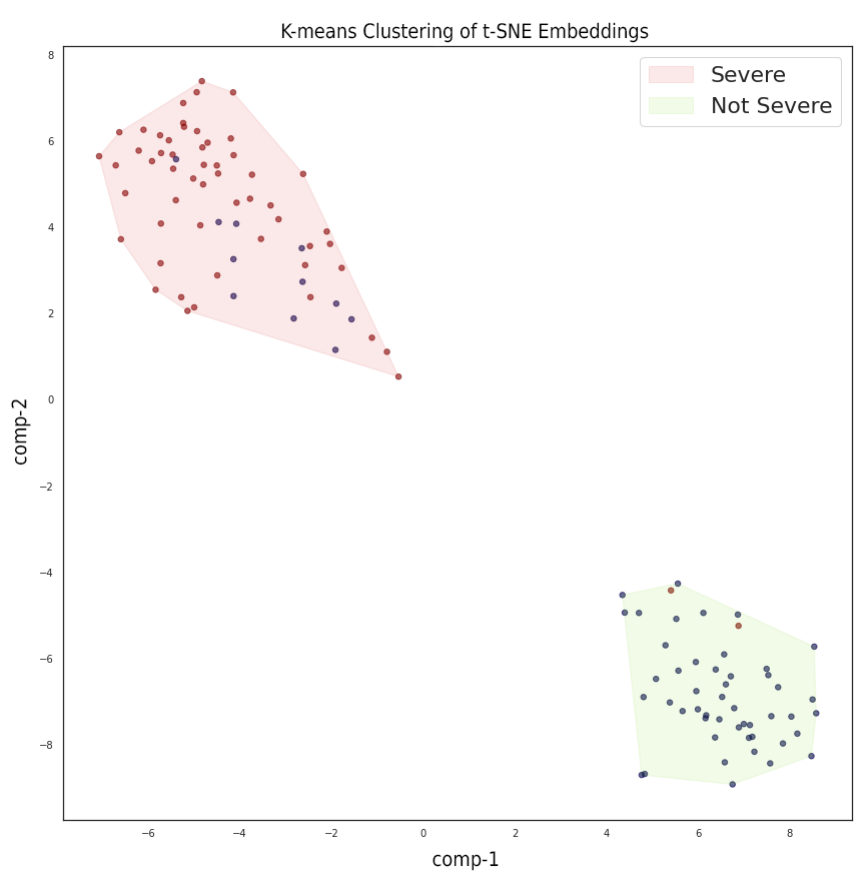
Visualizing the output of K-means clustering on test set t-SNEs. Note: The comp-1 and comp-2 axes denote the names of the 2 dimensions onto which t-SNE projects the 768D embeddings, where “comp” is short for “component”. t-SNE: t-distributed stochastic neighbor embedding.

Figure 1 visualizes the projected embeddings of our test queries before and after SBERT was fine-tuned using triplet loss. We found that SBERT learned meaningful clusters that largely separated severe from nonsevere samples. Using K-means clustering, we produced Figure 2, where the convex hull of each cluster is highlighted.

We were interested in analyzing patient queries that did not fall into the correct cluster. Textbox 1 highlights all the false positives, as determined by the K-means clusters shown in Figure 2. Specifically, these are the nonsevere (blue) samples in Figure 2 that appear in the Severe cluster.

A common theme among the false positives was that *all samples mentioned a symptom or disease*. SBERT was thus dependent on symptom interpretation for proper query embedding and likely misinterpreted how some symptoms were being presented. A qualitative analysis of Textbox 1 highlighted the following challenges:

- Symptom negation: Samples 1 and 4 highlight how negated symptoms may confuse telemedical triage symptoms. When analyzing sample 1, for example, a triage system must understand that the mentions of dry cough, fever, and sore throat are to highlight their absence and are not indicative of severity.
- Symptom temporality: Samples 4, 5, and 11 all have symptom mentions amidst complex temporal relationships. Automatic triage systems must be able to identify that not all symptom mentions are active, while highlighting what symptoms pertain to a given query.
- Ambiguous questions: Samples 2, 3, and 5 highlight a tricky subset of what we call “ambiguous questions,” where symptom mentions may occur but the purpose of the query is unclear or the proposed question is difficult to answer. Such samples were marked as not severe by the annotators.
- General queries: Samples 6, 7, 9, and 10 contain symptom mentions within the context of a general, nonsevere query. For example, the purpose of query 7, which came from a patient with pneumonia, was to obtain more information about how pneumonia manifests in the lung. This was not deemed severe by the annotators, as general-information requests should not be ranked higher over more relevant, specific, serious medical needs.
- Self-answered questions: Samples 8 and 11 contain valid explanations or resolutions to the problem being inquired about. For example, the patient in sample 11 had a continuous dry cough and sore throat. However, they had already taken all requisite COVID-19 precautions (COVID-19 test, self-quarantine). These samples were labeled not severe and may prove challenging for future telemedical triage systems.

Textbox 2 highlights all the false negatives, as determined by the K-means clusters shown in Figure 2. Specifically, these are the severe (red) samples in Figure 2 that appear in the Not Severe cluster.

A qualitative analysis of Textbox 2, which contains all false positives, identified other potential triage challenges:

- Sparse symptom representation: Sample 1 contains patient symptom mentions (boop, HIV, anemia), which contain little representation in the training data. As similar data sets expand the number of diseases for which they are able to triage, learning good representations of large symptom sets may prove challenging.
- Implicit symptom mentions: Sample 2 states they have all COVID-19 symptoms except fever. The reader understands this as meaning there is likely cough, loss of taste/smell, etc, present in the patient. SBERT is unable to make this inference, making this type of sample challenging.

In summary, SBERT’s errors are focused on samples with complex symptom presentation. Future work on telemedical triage may focus on explicit modeling of presented symptoms such that temporality, negation, intention, and other linguistic phenomena are accounted for.

**False-positive samples from the patient query test set.**

1. “I came into close contact with someone who just flown back from Australia, I have been in self isolation since he landed, am I'm not showing any symptoms (dry cough, fever, sore throat) what is the next protocol? Do I go into work?”
2. “I have been recently diagnosed w the flu (nose swab test done). I am 34 years old- ex-smoker (quit completely for 7 years now) however each year I get ‘walking pneumonia’ at least once. I'm almost certain I've got it again now. Anything I can do to stop getting pneumonia??”
3. “Hi! So I’m a 20 year old female. I started working out about a year ago. I noticed some lower abdominal pain after partaking in abdominal workouts. But also notice it around the time of my period. It’s right next to/under my hip bone on left side.”
4. “I recently got over either the flu or pnemonia. I've noticed my feces are increasingly yellow or whitish. I had quit smoking thrity days ago and have been on the nicotine gum and now the lozenges. I don't feel bad, but this is unusual. Could the nicotine products be contributing to the discoloration? Thank you.”
5. “Last year during flu season had a severe cough, difficulty breathing and xray show there was fluid in my chest/lungs. Any advice on what to do with this covid 19.”
6. “Hi. What medication can I take for sinus and headaches during this time of the virus? Thank you.”
7. “I was recently diagnosed with Pneumonia. I had pneumonia when I was a baby but never since. I was shocked when I heard the diagnosis. The symptoms became apparent on March 28. I began taking Doxycycline Monohydrate on Monday April 3 and must take them for seven days. I am mega healthy and have not been sick in years. I can t even remember the last time I was sick. This has really knocked me out. I have zero energy and very little appetite. How does pneumonia manifest in the lung and how does bacteria get in there? How long will it be until I recover? I am really having a hard time with this. Help!”
8. “Hi,my sinuses usually act up during seasonal change (like now). My worry is my symptoms resemble that of covid-19. Throat has been a bit irritated, lately nose has been runny. Wanted to know how I could get myself tested as I don’t live alone, thanks?”
9. “Throat a bit sore and want to get a good imune booster, especially in light of the virus. Please advise. Have not been in contact with Nyone with the virus.”
10. “Hello, I am a student and dealing with a microbiology assignment.I am given a paitent sample.My paitent is 4 years old -diagnosis Pneumonia - summary of peresent illness =Recurrant colds, ear infections,and bronchitis.She has been sick for past 3 weeks. Developed a fever yesterday.Also nausia and vomiting,muscle aches. Past Medical history= Cystic Fibrosis diagnosed at age 3. I did all the lab work and found out that the bacteria causes the disease is Psudomonas aeruginosa. What is the appropriate treatment? Please help.”
11. “Hi. This COVID-19 outbreak is scary. I got screened this week and it was negative. But prior to screening I had a week of continuous dry coughs and also throat was sore. I've put myself in a quarantine. What next? Do I still need to screen again?”

**False-negative samples from the patient query test set.**

1. “My dr. Did a routine CNBC last week. His nurse called and my blood showed signs of anemia. Ok today his nurse called and the blood they deeper searched on showed: the disease of chronic pneumonia? Ok I do not have hiv/AIDS. My question is she said no cure. My mom died from a chronic infection in her longs acronym Boop. I contacted cdc.gov they put me in touch with center for rare diseases. No cure for mom. Question: the told me Boop was not genetically transferred. This is exactly how my moms lung diseases started. is thimy lung disease genetic? Is it curable? Help please.”
2. “Good morning I have all the symptoms for the coronavirus except a high fewer. I have been in contact with someone (who now also are displaying these symptoms) who are staying with a person who visited India in the last few weeks. Should I be worried?”

## Discussion

### Principal Results

In this study, we provided a novel extension of the COVID-Dialogue data set with telemedical query severity labels. Further, we thoroughly investigated the capability of several transfer learning approaches to predict severity in a resource-constrained setting. We concluded that transformer-based models are able to triage with high efficiency (all F1 scores>0.9). Further, we provided a thorough error analysis of SBERT, highlighting challenging samples that require a deep understanding of symptom presentation. Our error analysis highlights various avenues for future work that explicitly model various patient query types.

This is a new area of research and requires more investigation to define the requirements of a real deployment. It should be noted that such systems should not be used for diagnosis. Such a solution can benefit from online learning approaches, especially in the context of the pandemic (eg, temporal and spatial factors are important for detecting outbreaks of a new variant of infection).

### Interpretability: Performance Trade-off

A commonly discussed limitation of deep neural networks (DNNs) is their lack of a natural way to explain the predictions they have made [39,40], so the use of DNNs makes it difficult to ask *why* a certain sample was predicted as severe or not severe. Models such as long short-term memory (LSTM) and transformer-based models make it challenging to identify when unfair biases or spurious correlations drive predictions. Thus, the use of transfer learning in telemedical triage must be done with care, as biases learned from other data sets may influence triage decisions.

Lexical models, such as TF-IDF, in combination with a linear classifier, provide straightforward access to a model’s utilization of certain vocabulary terms.

Given that our BERT-based models only provided up to a 5-point increase in the F1 score, we compared the test set errors of SBERT and TF-IDF+SVM to highlight specific sample types that require complexity and knowledge transferability of the transformer architecture for accurate prediction (Table 3).

Across all test sets (ie, the test set from each fold in 5-fold cross-validation), we found that the lexical model using TF-IDF made 77 errors, while SBERT made 47 errors. Additionally, 81% of the errors made by SBERT were also made by TF-IDF. Of the 39 samples predicted correctly by SBERT but incorrectly by TF-IDF, we highlighted 7 queries in Table 3 that are representative of the TF-IDF errors. A qualitative analysis of Table 3 highlighted the following:

- General queries: These samples either inquire about general medical knowledge or request information about COVID-19 testing from a nonsymptomatic patient. Samples 1 and 2 highlight an example of each general query type. These samples were challenging for TF-IDF, given lexical models may struggle to understand query intent without contextual modeling, as our TF-IDF model only considers unigram features. From our 39-sample evaluation set, 22 predictions made by TF-IDF were false positives, with 59% of them being on general query samples.
- Ambiguous questions: These samples are queries that do not contain enough information for a valid response or do not pose a question that can benefit from a remote physician. Samples 3 and 4 are examples of ambiguous questions. We found that 15% of the TF-IDF errors that SBERT predicted correctly are predictions on ambiguous questions.

Many of the false negatives had no obvious content-based justification for TF-IDF’s failure. In other words, TF-IDF’s issue with the false negatives in Table 3 appear to be due to symptom sparsity and spelling errors. The ability of transformer-based models to transfer knowledge and analyze subwords makes it better suited for such samples, which are realistic issues to be faced by any telemedical triage system put into production.

**Table 3:** Subset of samples predicted incorrectly by TF-IDF^a^+SVM^b^ but predicted correctly by SBERT^c^.

Sample number	Patient query	Ground truth label
1	“About the ibuprofen and covid 19 should I quit taking it? It's got me paranoid. The way the media's been talking about it. I take it everyday for my neck pain and back pain. I can't take pain pills because they make me nauseas. Any insight please”	0
2	“Hi, I arrived from the Netherlands on Monday morning. No symptoms but have been around my helper. Should we get tested”	0
3	“I'm finding difficult to maintain precisely 6 ft in grocery stores. Today, as I was leaving, someone entering the store that was (possibly) 3 ft away was coughing lightly, and I took a shower when I got home. I'm a hypochondriac. Possible covid-19?”	0
4	“Hi, My uncle has been diagnosed with liver cancer and he is in the last stage. After the first chemotherapy he has been admitted to hospital due to pneumonia. Is he again diagnosed with lung cancer? And what are the chances of getting cure? What treatment you would like us to get it done.”	0
5	“I believe I might have Covid 19 symtoms. It's possible to get testing done at home to confirm? Currently I have soar throat, started last night around 19:30.”	1
6	“Hi my husband has been puking since this morning, has serious vertigo + is off balance. Im suspecting food poisoning but want to be sure. I gave him a pill for nausea, which is working. Do I still take him to a doctor to check that its nothing else?”	1
7	“I live in france.and now 7days for home quarantine.i have no fever.but I have parangities in my thoart. last few years it's comes and goes. now I am worried because of covid-19. Does only parangities is only symptoms of this???”	1

^a^TF-IDF: term frequency–inverse document frequency.

^b^SVM: support vector machine.

^c^SBERT: sentence bidirectional encoder representation from transformers.

### Limitations and Future Work

We were unfortunately only able to look at telemedical triage through the lens of patients with COVID-19 or related pneumonia symptoms. Real-world systems will need to understand a diverse set of diseases and symptoms to handle the variance in queries doctors will receive. For example, HealthTap offers medical advice in over 147 specialties, necessitating a system with a deeper understanding of different medical conditions. In the future, we plan to extend our system so that it can classify patient queries that span a more diverse set of medical conditions.

Like any other automatic recommendation system, the performance of such an automated triage system might be affected based on the quality of user queries. For instance, an automatic triage system can assign lower severity to queries that have missing information (ie, a patient forgets to mention relevant symptoms or does not share enough details) or are not well written. This is similar to Google Search, where the quality of search results depends on the user query and where user satisfaction is correlated with the quality of the query. Such automatic triage can still be useful and significantly improve longitudinal user interaction at scale, as has been shown in other recommendation systems (eg, Google Search or Amazon recommendations). Another limitation is the binary classification system, as it ignores the spectrum of potential perceived severity. Future work could develop a score-based system where severity is scored on a continuous scale. Further research with multidisciplinary research teams is required to determine the impact of such automated solutions and identify potential techniques to address such limitations.

In future work, we will extend this system beyond the online medical Q&A forum and into doctor-patient messaging apps. We are actively in conversation with a local teaching hospital regarding the issue of doctors being overwhelmed with textual medical queries. Thus the NLP models explored in this paper may prove useful in future works modeling doctor-patient messaging data, including tasks such as the relative ranking of patient query importance of in-hospital private messaging systems. However, the triage problem becomes more challenging in the hospital messaging system context as patients will naturally assume their doctor is familiar with their medical history, providing narrow and incomplete information in their textual queries. To handle this problem, future systems must be able to make inferences using multiple modalities (eg, EHRs, medical images), as well as past conversations, which will likely require a shift in deep learning architecture as BERT-based models are restricted to processing of 512 tokens.

### Conclusion

Telemedical triage is an important task in the world of telemedicine. Ranking medical queries according to severity both optimizes the doctor’s time and allows care to be administered to more of those with time-sensitive issues. We showed that even in the presence of a limited amount of data, transfer learning can be used to triage for COVID-19 and pneumonia patients with high accuracy. Specifically, we found a statistically significant difference in performance between transformer-based solutions and both lexical and GloVe embedding-based solutions. We additionally categorized all model errors into numerous interpretable categories, highlighting sample types that challenge our NLP-based triage systems. Queries with complex negation, temporality, and ambiguity (among other linguistic phenomena) were shown to be highly present in SBERT’s errors, giving specific direction for future work on telemedical triage.

## Abbreviations

AI: artificial intelligence
BERT: bidirectional encoder representation from transformers
bi-LSTM: bidirectional long short-term memory
DNN: deep neural network
ED: emergency department
EHR: electronic health record
GloVe: global vectors for text representation
HAN: hierarchical attention network
KNN: K-nearest neighbor
LSTM: long short-term memory
NLP: natural language processing
Q&A: question-and-answer
RQ: research question
SBERT: sentence bidirectional encoder representation from transformers
SVM: support vector machine
TF-IDF: term frequency–inverse document frequency
t-SNE: t-distributed stochastic neighbor embedding

## Data Availability

Data and code from this study can be found on GitHub.
